# Complement Factor C7 Contributes to Lung Immunopathology Caused by *Mycobacterium tuberculosis*

**DOI:** 10.1155/2012/429675

**Published:** 2012-07-30

**Authors:** Kerry J. Welsh, Cole T. Lewis, Sydney Boyd, Michael C. Braun, Jeffrey K. Actor

**Affiliations:** ^1^Department of Pathology and Laboratory Medicine, University of Texas Medical School at Houston, Houston, TX 77030, USA; ^2^Department of Pediatrics-Renal Section, Texas Children's Hospital, Baylor College of Medicine, Houston, TX 77030, USA

## Abstract

*Mycobacterium tuberculosis* (MTB) remains a significant global health burden despite the availability of antimicrobial chemotherapy. Increasing evidence indicates a critical role of the complement system in the development of host protection against the bacillus, but few studies have specifically explored the function of the terminal complement factors. Mice deficient in complement C7 and wild-type C57BL/6 mice were aerosol challenged with MTB Erdman and assessed for bacterial burden, histopathology, and lung cytokine responses at days 30 and 60 post-infection. Macrophages isolated from C7 −/− and wild-type mice were evaluated for MTB proliferation and cytokine production. C7 −/− mice had significantly less liver colony forming units (CFUs) at day 30; no differences were noted in lung CFUs. The C7 deficient mice had markedly reduced lung occlusion with significantly increased total lymphocytes, decreased macrophages, and increased numbers of CD4+ cells 60 days post-infection. Expression of lung IFN-*γ* and TNF-*α* was increased at day 60 compared to wild-type mice. There were no differences in MTB-proliferation in macrophages isolated from wild-type and knock-out mice. These results indicate a role for complement C7 in the development of MTB induced immunopathology which warrants further investigation.

## 1. Introduction

 Tuberculosis (TB) remains a significant public health problem, causing nearly 2 million deaths each year [1]. Host protection against TB requires the development of a Th1 immune response [2]. Th1 responses are initiated by dendritic cell presentation of MTB antigen to naive CD4+ T-cells accompanied by synthesis of IL-12 [3]. These Th1 cells produce IFN-*γ*, which activates macrophages to cause phagosome acidification, phagolysosome fusion, and the generation of reactive nitrogen species that control MTB growth [2]. It is essential to understand the innate immune system functions that lead to protective T-cell responses to MTB in order to develop improved vaccines and therapies based on immune modulation. 

 Increasing evidence indicates that the complement system is essential for generating protective adaptive immune responses to mycobacterial infections [4–6], in addition to its clear role in both innate immunity and the initiation of adaptive immunity to a variety of other pathogens [7]. The three major pathways of complement are the classical, alternative, and lectin pathways. Each complement pathway leads to the cleavage of C3 generating C3a and C3b. C3b binding to pathogens promotes opsonization resulting in the enhanced elimination pathogens, as well as the formation of C5b-9, the membrane attack complex. Insertion of the complement component C7 into the cell membrane is the critical step in the formation of the membrane attack complex that leads to cell lysis. 

 The precise role of complement factors in immunity and pathology during mycobacterial infection is unclear. It was reported that mice C3 knockout mice infected with *M. avium* had no differences in granuloma formation or bacterial load in comparison to wild-type mice [8]. However, mice deficient in complement C5 have decreased survival, increased inflammation, and decreased macrophage cytokine production [4, 5, 9]. Little is known about the role of the terminal complement components in MTB infection. Thus, complement factor C7 knock-out (C7 −/−) mice were infected with MTB and evaluated for bacterial burden, lung immunopathology, and cytokine responses. 

## 2. Methods

### 2.1. Animals 

 Six week-old C7 deficient mice (C7 −/−) and wild-type C57BL/6 mice were a kind gift from Michael C. Braun (Baylor College of Medicine, Houston, TX). Briefly, C57BL/6J ES cells with a targeted insertion of a gene trap vector in the intronic region between exons 6 and 7 of the C7 gene were obtained from the Texas Institute of Genomic Medicine (TIGM, Houston, Texas). Single insertion of the vector was confirmed by FISH analysis, and targeting to the intronic region between exons 6 and 7 of the C7 gene was confirmed by sequence analysis. Heterozygous chimeric mice with germ-line integration were then back-crossed with C57BL/6J mice. The successful gene trapping of C7 using the targeting vector was confirmed by RT-PCR, and hemolytic assay. Six to eight mice were used per group, per time points indicated, infected within biosafety level 3 facilities. All studies were performed under the approval of the Animal Welfare Committee at UTHSC, protocol AWC-11-020. 

### 2.2. Acute Tuberculosis Infection of Mice

 MTB strain Erdman (TMC 107, American Type Cell Culture) was cultured in Middlebrook 7H9 broth, with 10% supplement, to log phase. Pelleted bacteria were resuspended in phosphate buffered saline (PBS) and diluted to 3 × 10^8^ colony forming units (CFU) per mL using McFarland standard #1 (Thermo Fisher Scientific-Remel, Lenexa, KS). Bacteria were sonicated to disperse any clumps. C57BL/6 mice were infected using an aerosol inhalation exposure system (GLAS-COL Model #A4212 099c, Glas-Col, Terre Haute, IN) to achieve an average aerosol implantation of 100 CFUs, as described [10]. The inoculation dose was confirmed by sacrificing a subset of mice at day one after the-challenge and plating lung homogenates onto 7H11 agar plates (Remel, Lenexa, KS), which were incubated at 37°C for 3-4 weeks.

 Mice were sacrificed at days 30 and 60 after the-MTB challenge. The lung, liver, and spleen were removed and sectioned. Portions used for determination of CFUs were placed into 5 mL PBS and homogenized. The organ homogenates were serial diluted and plated on Middlebrook 7H11 plates. Colonies were enumerated after a 3-4 week incubation period at 37°C. 

### 2.3. Lung Histopathology Analysis

Lung tissue was fixed in 10% formalin and embedded in paraffin. Five *μ*m thick sections were stained with hematoxylin and eosin by standard methods. Acid-fast staining was performed by the Ziehl-Neelsen method. Six lungs from each group at 30 and 60 days post-challenge were randomly selected for immunohistochemistry (IHC) for CD4 and CD8. Rat anti-mouse monoclonal antibodies (R&D Systems, Minneapolis, MN) were used to detect CD4+ and CD8+ cells using an anti-rat horseradish peroxidase 3,3′-diaminobenzidine cell and tissue staining kit according to the manufacturer's instructions (R&D Systems).

 Histopathology images were obtained with the Nuance multispectral imaging system (CRI, Woburn, MA). This system allows the enumeration of cell types in defined areas of pathology. The entire section of the immunostained lungs was imaged using the 10x objective. Quantification of normal lung, area percentages of macrophages and lymphocytes, and total number of CD4+ and CD8+ cells was performed using the tissue and cell segmenting functions of Inform software (CRI) using the manufacturer's instructions.

### 2.4. Lung Cytokine Expression

Total lung RNA was isolated and used to produce cDNA as previously detailed [11, 12]. Analysis of cDNA was performed using qRT-PCR procedures, with previously detailed primers and probes [12]. The reaction mixture contained 5 *μ*L cDNA, 1X PCR buffer (5Prime, Fisher Scientifics, Pittsburg, PA), 200 nM dNTPs (Invitrogen, Carlsbad, CA), 400 nM each for the forward and reverse primer (Integrated DNA Technologies, San Diego, CA), 100 nM probe (Biosearch Technologies, Novato, CA), 1x ROX reference dye (Invitrogen), and 1 U/50 *μ*L Taq DNA polymerase (Fisher). The reaction was performed using the ABI Sequence Detection System (Applied Biosciences, Carlsbad, CA) by heating for 1 min at 95°C, and then 40 cycles of 95°C for 12 seconds and 60°C for 1 min. Data are expressed as fold change expression relative to naive controls after normalization to *β*-actin [13].

### 2.5. Preparation of Bone-Marrow-Derived Macrophages and Infection with MTB

Bone-marrow-derived macrophages from wild-type C57BL/6 and complement factor C7 −/− mice were generated by previously described methods [14]. Briefly, femurs were flushed with McCoy's media (Sigma, St. Louis, MO), and 2 × 10^6^ cells were added to 24 well tissue culture plates (Corning Incorporated, Corning, NY). Cells were cultured in McCoy's media (Sigma) supplemented with 10% fetal bovine serum (FBS) (Sigma), 100 *μ*g mL^−1^ gentamycin (Sigma), 100 U mL^−1^ penicillin (Sigma), and 10 ng mL^−1^ recombinant murine granulocyte/macrophage colony stimulating factor (GM-CSF; Chemicon, Billerica, MA). The cells were cultured at 37°C in 5% CO_2_ for seven days, with two media changes containing GM-CSF. Finally, cells were washed and suspended in antibiotic free Dulbecco's modified eagle's medium (DMEM; Sigma) supplemented with 10% FBS. 

The cells were infected with log phage cultures of MTB Erdman at a multiplicity of infection (MOI) of 1 : 1. The supernatants were removed after 24 and 72 hours of incubation and analyzed for production of IL-6, TNF-*α*, IL-12p40, and TGF-*β* by ELISA according to the manufacturer's instructions (R&D Systems).

### 2.6. Statistics

The data are shown as the mean ± SD. Two-way ANOVA was used to determine the differences between groups by use of GraphPad Prism software. A *P*-value of less than 0.05 was defined as statistically significant.

## 3. Results

### 3.1. Decreased Bacterial Dissemination in C7 Deficient Mice 

**Figure 1:**
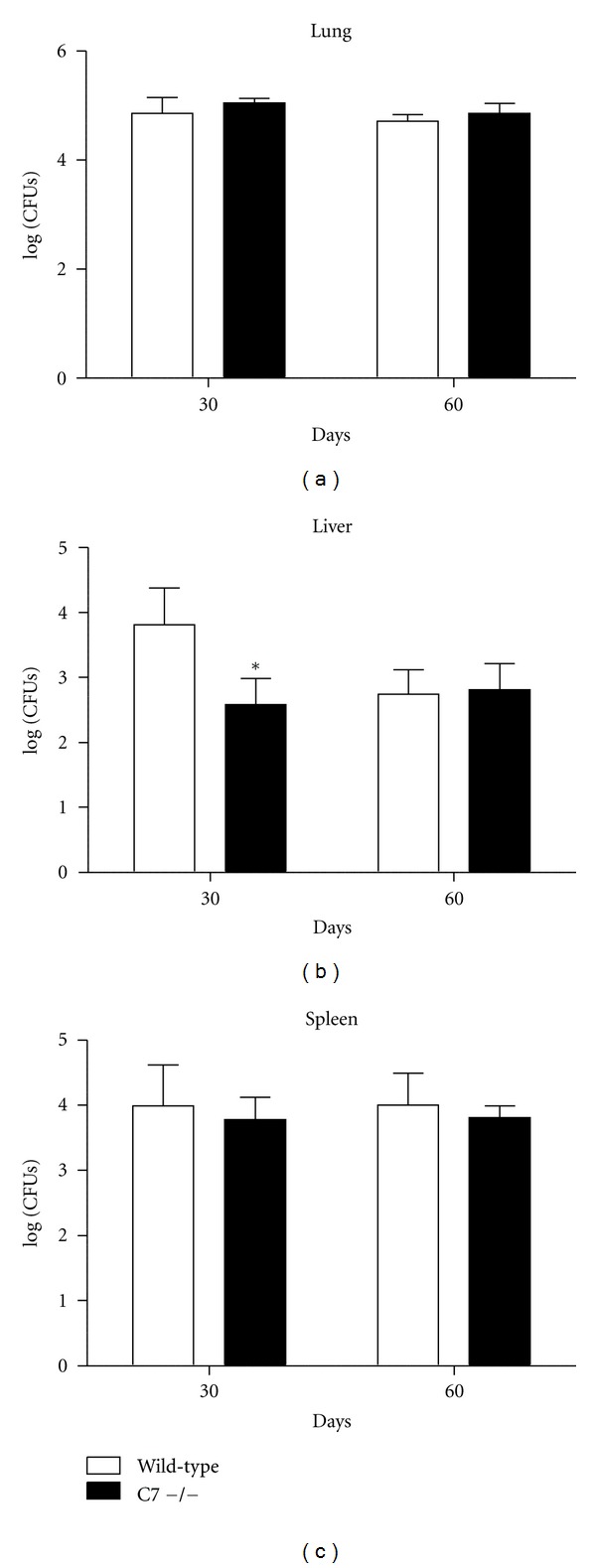
Decreased early bacterial dissemination to the liver in C7 −/− mice. C7 −/− mice had significantly reduced bacterial CFUs in the liver at day 30 after infection with MTB. There were no differences in bacterial burden in the lung or spleen. *P < 0.05 with comparison to wild-type C57BL/6 mice.

 Bacterial CFUs in the lung, liver, and spleen are shown in Figure 1. C7 −/− mice had significantly reduced CFUs in the liver at day 30; specifically, liver log_10_ CFUs in wild-type mice were 3.82 ± 0.56 and 2.58 ± 0.40 at day 30 for the C7 −/− mice. No significant differences in bacterial load were observed in the lung and spleen at either time point.

### 3.2. C7 −/− Mice Have Decreased Lung Immunopathology

**Figure 2:**
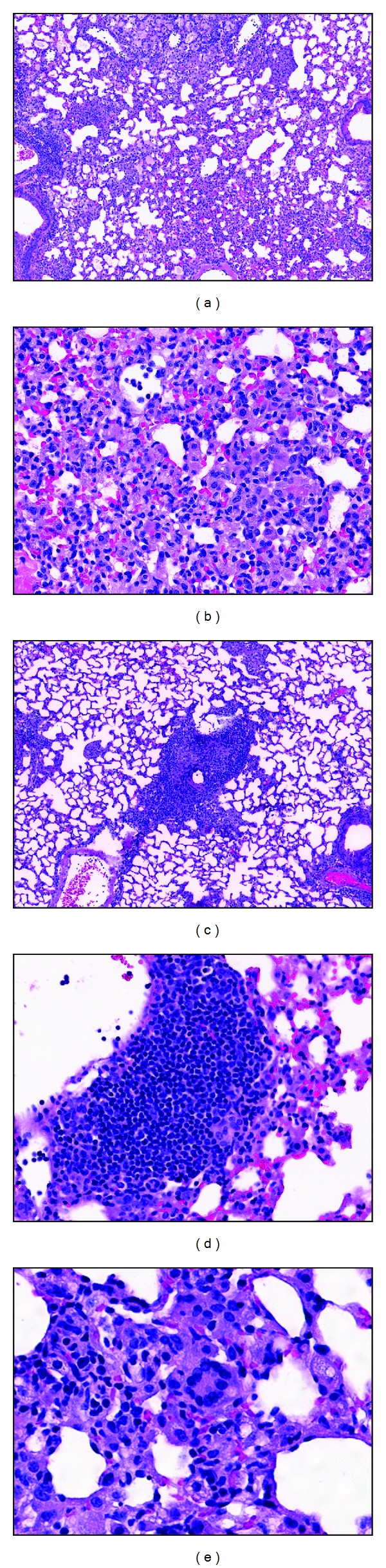
Lung histopathology of wild-type C57BL/6 and C7 −/− mice after the-challenge with MTB Erdman. (a) C57BL/6 mice demonstrate extensive granulomatous inflammation at 60 days post infection, 100x. (b) High power images of wild-type mice show that infiltrates are largely macrophages, 400x. (c) C7 −/− mice have reduced lung immunopathology at day 60 after the infection, 100x. (d) Abundant areas with clusters of lymphocytes are observed in C7 −/− mice, 400x. (e) Rare multinucleated giant cell in C7 −/− mice, 400x.

 Representative images of lung histopathology for wild-type and C7 −/− mice at 60 days post-infection are shown in Figure 2. Wild-type mice demonstrate significant lung inflammatory infiltrates, which were predominantly macrophages (Figures 2(a) and 2(b)). In contrast, C7 −/− mice have reduced destructive lung pathology with smaller granulomas and decreased parenchymal inflammation (Figure 2(c) and Table 1). Abundant clusters of lymphocytes were observed in the C7 −/− mice, primarily near the vasculature and adjacent to granulomas (Figure 2(d)). Quantitative IHC demonstrated increased numbers of CD4+ lymphocytes and reduced areas of macrophages in the C7 −/− mice (Table 1). Rare multinucleated giant cells were noted in the C7 −/− mice (Figure 2(e)).

### 3.3. Absence of Complement Factor C7 Modulates Lung Cytokine Expression 

**Figure 3:**
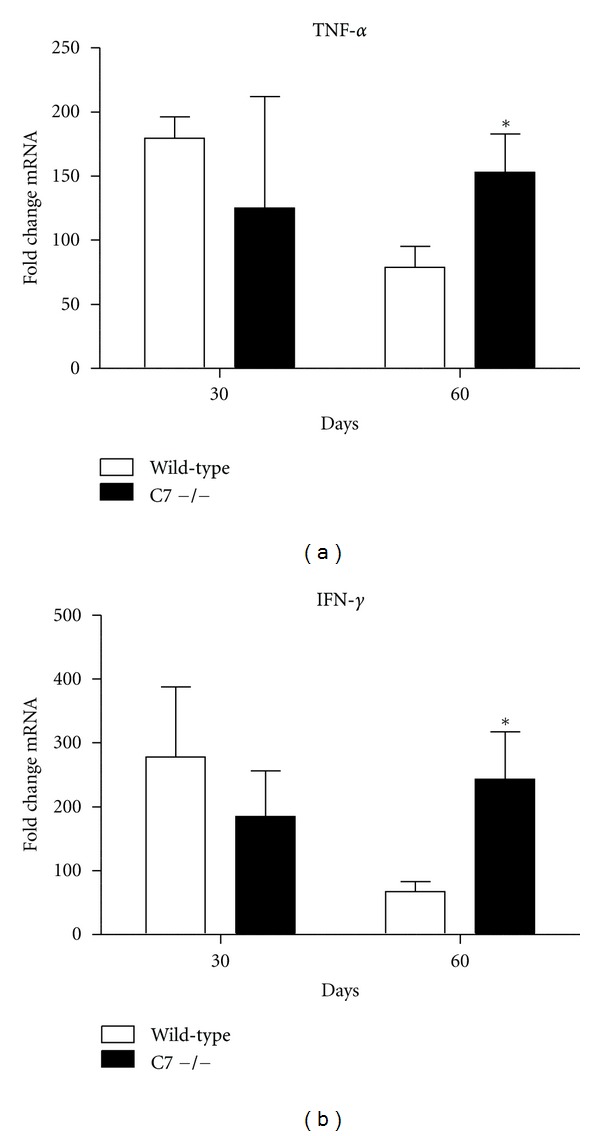
Lung expression of proinflammatory mediators TNF-*α* and IFN-*γ* in wild-type and C7 −/− mice. Lung cytokine expression of TNF-*α*, and IFN-*γ* as measured by quantitative PCR in wild type (solid bars) and C7 −/− (open bars) mice at 60 days post challenge with virulent MTB. Data are expressed as fold change relative to naïve mice after normalization to *β*-actin. Data are presented as the mean with SD, *n* = 6 mice per group, per time point. *P < 0.05, comparisons are made to control mice. There were no significant differences in lung expression of IL-6, IL-12p40, TGF-*β*, or IL-17 (data not shown).

Lung cytokine expression of TNF-*α*, IL-6, IL-12p40, TGF-*β*, IL-10, IFN-*γ*, and IL-17 was measured by quantitative PCR (Figure 3). Expression of all cytokines increased in challenged wild-type mice relative to naive mice. C7 −/− mice had significantly increased expression of TNF-*α* and IFN-*γ* at day 60 after the-MTB challenge, compared to wild type mice. There were no significant differences in lung expression of IL-6, IL-12p40, TGF-*β*, or IL-17 (data not shown).

### 3.4. Modulation of Macrophage Cytokine Production by Complement C7 

**Figure 4:**
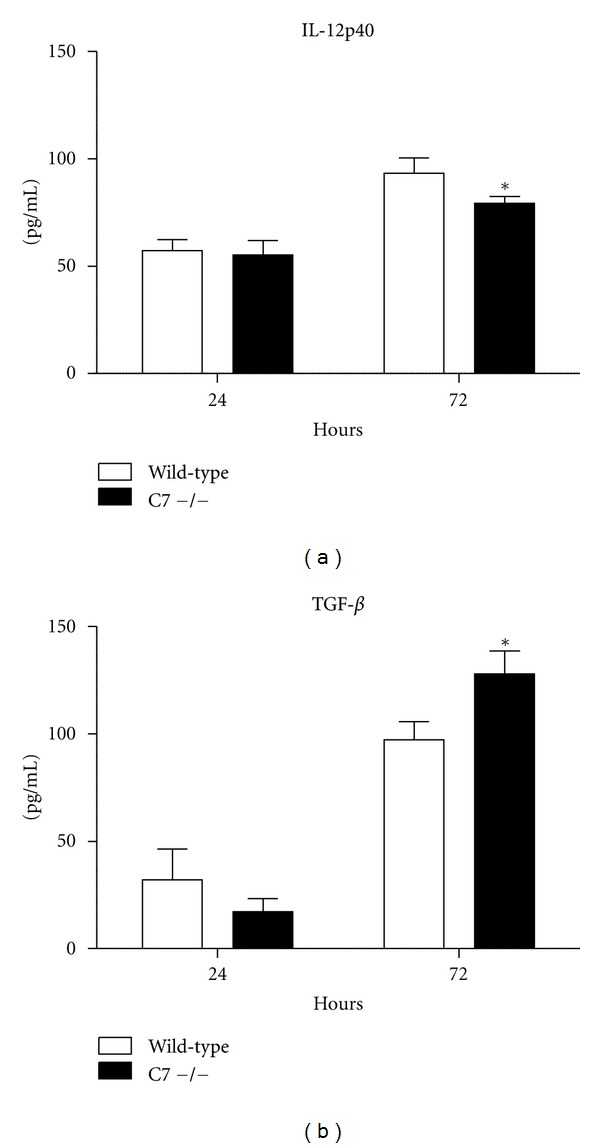
Cytokine production from infected bone-marrow-derived macrophages. Macrophages were isolated from wild-type and C7 −/− mice and infected with MTB at a MOI of 1 : 1. Cytokines production was measured 24 and 72 hours after infection by ELISA. *P < 0.05, comparisons are made to control mice.

 Proinflammatory cytokine production was measured from MTB-infected bone-marrow-derived macrophages isolated from wild-type and C7 −/− mice (Figure 4). The macrophages from the C7 −/− mice had slight, but significant, decreased synthesis of IL-12p40 and increased TGF-*β* at 72 hours post-infection. There were no differences in synthesis of TNF-*α* and IL-6 (not shown). Additionally, the absence of complement factor C7 did not affect MTB proliferation in macrophages (not shown).

## 4. Discussion

 While deficiencies in the terminal complement factors such as C7 are associated with *Neisseria meningitidis *infections [15], their function in mycobacterial infections is unknown. These studies indicate a role for complement C7 in the development of lung immunopathology caused by infection with MTB. Mycobacteria are known to activate all three complement pathways [16], but the results of specific pathway activation on MTB pathogenesis are mixed. While complement activation likely promotes the uptake of mycobacteria into macrophages [8, 17], deficiency of C5 resulted in enhanced susceptibility to TB and its associated trehalose 6,6′-dimycolate glycolipid; this was evident by a reduced IL-12 production by macrophages and reduced IFN-*γ* synthesis and production [6, 9]. Prior investigations demonstrate that macrophages and dendritic cells produce complement C7 [18, 19]; we therefore determined the impact of complement C7 deficiency on MTB-infected macrophage cytokine secretion. A slight reduction in IL-12p40 and an increase in TGF-*β* were noted, possibly indicating that C7 deficiency may impact induction of T-cell responses. 

 The complement C7 deficient mice had increased expression of IFN-*γ* and TNF-*α* at 60 days post-challenge with MTB accompanied by increased numbers of CD4+ lymphocytes, despite a decrease in IL-12p40 by infected macrophages. Numerous investigations link the early complement cascade components to the development of Th1 responses [20]. For example, the binding of complement factors C3a and C5a to their receptors promotes the migration of antigen presenting cells to areas of infection, where they present antigen to T-cells and modulate IL-12 production that is essential for induction of IFN-*γ* synthesis [21–23]. Binding of immune complexes to the C1q receptor on T-cells promotes T-cell activation with production of TNF-*α* and IFN-*γ* [24]. Both CD4+ and CD8+ T-cells responses in C3 deficient mice are decreased in viral infection models [25–27]. The precise role of complement C7 in induction of IFN-*γ* synthesis is unclear; however, there are reports of CD59 modulation of T-cell responses. These data suggest that the crosslinking of CD59, an inhibitor of MAC formation at the level of C9, resulted in augmented IL-2 production and cell proliferation [28]. Furthermore, CD59a deficient mice have enhanced specific CD4+ T-cell responses after challenge with recombinant vaccinia virus [29]. It should be noted that the increase in lung expression of IFN-*γ* in the C7 −/− mice did not result in a decrease in lung bacterial burden; only an early reduction in liver CFUs was observed. Some investigators report little correlation between the levels of specific IFN-*γ* producing CD4+ and CD8+ cells and protection against MTB [30]. 

 Of potential significance, the C7 deficient mice had markedly reduced lung immunopathology in this mouse model of MTB infection. The histopathology generated by the host in response to MTB possibly contributes to the persistence and dissemination of the organism [31]. For example, MTB in granulomas convert to a state of non-replicating persistence that is characterized by alterations in biochemical pathways and reduced bacterial proliferation, changes that make the bacteria resistant to antibiotics [32, 33]. The altered cytokines in the infected C7 −/− mice are clearly important in granuloma induction and maintenance of structure [12]. MTB contained with granulomas are physically sequestered from cytotoxic T-cells capable of eliminating infected cells [31]. Reduction of lung immune-mediated pathology may additionally enhance the penetration of antibiotics into infected tissue, possibly enabling a more rapid response to antimicrobials [34]. Indeed, modulation of this pathology has been proposed as a novel therapeutic strategy to shorten the treatment of TB, an approach that has had some success in human clinical trials [35, 36]. Future studies are needed to explore the therapeutic potential of C7 on MTB-induced pathology. 

 The data presented in these studies indicate a role for complement C7 in the development of lung histopathology in MTB infection, with a potential role for dysregulation in innate interaction with lymphocytes and their subsequent production of cytokines. Future studies are needed to further investigate the role of C7, to specifically define the direct link between the regulation of response affecting MTB-induced immunopathology. In this manner, we can further understand the relationship of pathological dysregulation directly attributed to C7 compared to its contribution as related to other terminal complement components.

## Figures and Tables

**Table 1:** Quantitative IHC of wild-type C57BL/6 mice and C7 deficient mice infected with MTB.

	Control	C7 −/−
% lung occlusion		
Day 30	50.6 ± 12.1	47.8 ± 12.3
Day 60	59.1 ± 12.8	40.8 ± 7.5*
% macrophages		
Day 30	50.8 ± 12.0	39.9 ± 8.1
Day 60	49.2 ± 13.1	30.3 ± 10.1*
% lymphocytes		
Day 30	4.3 ± 2.8	6.7 ± 3.7
Day 60	9.1 ± 3.1	17.0 ± 8.7*
CD4+ lymphocytes		
Day 30	277.2 ± 122.9	348.9 ± 239.5
Day 60	291.1 ± 155.9	973.9 ± 449.2*
CD8+ lymphocytes		
Day 30	321.3 ± 177.1	417.3 ± 169.8
Day 60	422.3 ± 284.8	440.3 ± 216.4
